# Complex Effects of B-Vitamin Combinations on Cardiovascular Diseases: A Systematic Review and Meta-Analysis of Randomized Controlled Trials over Three Decades

**DOI:** 10.3390/nu18050842

**Published:** 2026-03-05

**Authors:** Ruodi Ren, Andrew Yang, Allison Chow, Kunkun Wang, Shan Wang, Christopher Leo, Yun Lu, Mengyan Li

**Affiliations:** 1 College of Pharmacy, University of Minnesota, Minneapolis, MN 55415, USA; renruodicc@gmail.com; 2 Dartmouth College, Hanover, NH 03755, USA; andrew.k.yang.21@dartmouth.edu; 3 College of Arts and Science, New York University, New York, NY 10012, USA; ayc8022@nyu.edu; 4 University of Washington School of Medicine, 1959 NE Pacific St., Seattle, WA 98195, USA; kvwang@uw.edu; 5 NYU Langone Hospital—Long Island, Mineola, NY 11501, USA; shan.wang@nyulangone.org; 6 Duke Raleigh Hospital, a Campus of Duke University Hospital, School of Medicine, Duke University, Durham, NC 27708, USA; christopher.leo@duke.edu; 7 College of Pharmacy, University of Minnesota, Hennepin Healthcare System, Minneapolis, MN 55415, USA; yunlu@umn.edu; 8 Department of Pharmacy, Hennepin Healthcare System, Minneapolis, MN 55415, USA; 9 Brigham and Women’s Hospital, Boston, MA 02115, USA

**Keywords:** stroke, myocardial infarction, mortality, folic acid, vitamin B12, vitamin B6

## Abstract

**Background and Purpose:** The effects of B-vitamin combinations on the prevention of cardiovascular diseases, such as myocardial infarction (MI) and stroke, remain controversial. We conducted a systematic review and meta-analysis of randomized controlled trials (RCTs) over three decades to evaluate the association between B-vitamin combinations and mortality and arterial thrombotic outcomes. **Methods:** PubMed, Embase, Web of Science, and the Cochrane Library were systematically searched for RCTs with minimal duration over 24 months published between January 1996 and November 2025. Two reviewers independently screened studies, extracted data, and assessed risk of bias using the Cochrane Risk of Bias 2.0 tool. Random-effects models were used in this meta-analysis to calculate pooled risk ratios (RRs) and 95% confidence intervals (CIs). **Results:** Thirteen randomized trials enrolling 68,363 participants across both primary and secondary prevention populations were included. B-vitamin combinations were associated with a nonsignificant reduction in stroke and 3-point major adverse cardiovascular events (MACE) (stroke: RR 0.91, 95% CI 0.81–1.04; MACE: RR 0.93, 95% CI 0.86–1.01). No significant effects were observed for all-cause mortality (RR 1.01, 95% CI 0.96–1.06), cardiovascular mortality (RR 0.97, 95% CI 0.88–1.07), or MI (RR 0.97, 95% CI 0.91–1.03). In primary prevention populations, B-vitamin combinations were associated with significant reductions in stroke (RR 0.79, 95% CI 0.68–0.93) and MACE (RR 0.80, 95% CI 0.69–0.92). A modest reduction in MACE was also observed in secondary prevention populations (RR 0.91, 95% CI 0.83–0.99). Between-study heterogeneity was minimal to low for ischemic outcomes, supporting the robustness of these estimates, whereas substantial heterogeneity was observed for mortality outcomes in secondary prevention populations. **Conclusions**: The evidence is limited by heterogeneity in trial populations, vitamin formulations and doses, and outcome definitions, with substantial between-study inconsistency for mortality outcomes and imprecision in subgroup estimates derived from a small number of contributing trials. Overall, B-vitamin combinations do not confer consistent benefit for major cardiovascular outcomes but may reduce stroke and MACE in selected primary prevention populations, suggesting that baseline cardiovascular risk and regional folic acid fortification modify treatment effects and should guide future trial design and clinical use.

## 1. Introduction

Elevated homocysteine levels, or hyperhomocysteinemia, have been found to be associated with increased risks of arterial thrombotic events, including ischemic stroke and myocardial infarction. Evidence from in vivo and in vitro studies suggests that hyperhomocysteinemia promotes platelet activation and oxidative stress, enhances endothelial dysfunction, and leads to a prothrombotic state [1,2]. Homocysteine is metabolized via vitamin B9- and B12-dependent remethylation and vitamin B6-dependent transsulfuration pathways [3]. Vitamins B6, B9, and B12 are key cofactors in homocysteine metabolism and have therefore been proposed as a strategy to reduce thrombotic risk [4].

Unfortunately, the protective effects of B-vitamin combinations on arterial thrombotic outcomes have been inconsistent across randomized trials [3], despite it possibly lowers homocysteine levels [5,6,7]. These discrepancies may be attributed to differences in baseline homocysteine levels, supplementation forms and dosages, geographic locations due to national folate fortification policies, genetic factors, concomitant medication use, and other underlying risk factors such as age, smoking status, and impaired renal function. Secondly, the trials have various primary objectives targeted either primary or secondary prevention and enrolled patients with different risk profiles. The use of diverse and heterogeneous outcomes—ranging from myocardial infarction and stroke to all-cause mortality or composite measures—can obscure true treatment effects in particular subgroups. Additionally, the extent of homocysteine reduction as well as the optimal combination and dose of B vitamins remain uncertain in various clinical trials. Therefore, inconsistent findings were seen when comparing the related studies.

Despite these inconsistencies, a consensus is still anticipated to guide the use of B-vitamin combinations across different clinical settings. The goal of this meta-analysis on the clinical trials from the past 30 years was to quantitatively determine the effects of B-vitamin combinations on arterial thrombotic outcomes and all-cause mortality. We also performed subgroup analyses according to outcome types, primary vs. secondary prevention, and relevant clinical characteristics to better evaluate the potential modifiers.

## 2. Materials and Methods

### 2.1. Search Strategy

**Figure 1:**
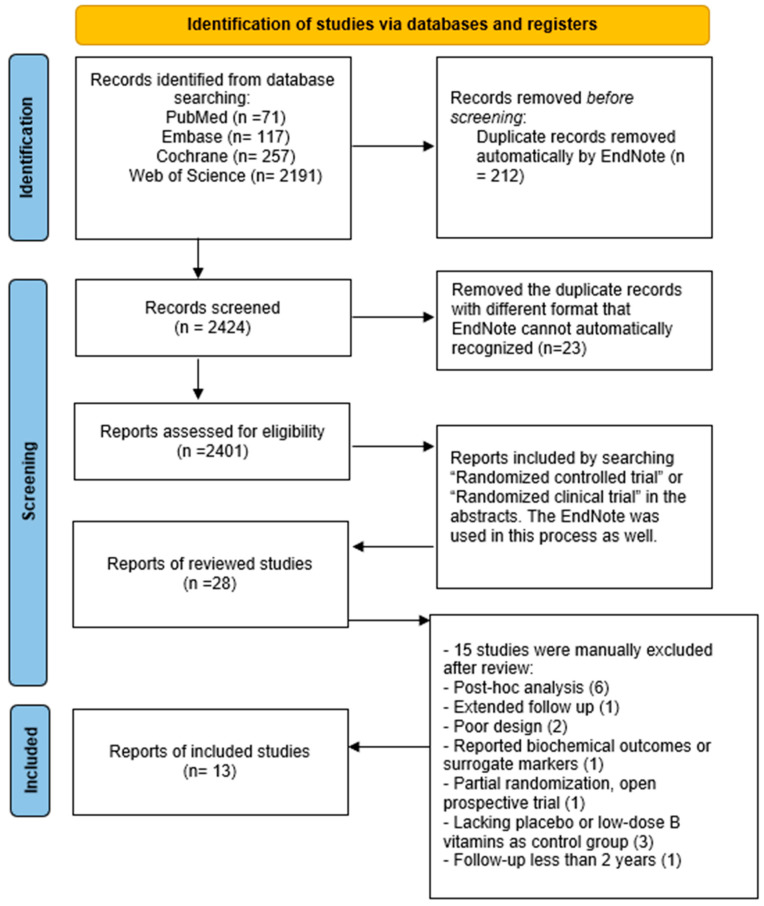
PRISMA 2020 flow diagram [8]. The diagram illustrates the process of study identification, screening, eligibility assessment, and inclusion in the meta-analysis, following the PRISMA 2020 guidelines.

A literature search was conducted using four databases (PubMed, Embase, Web of Science, and Cochrane) to identify all randomized controlled trials (RCTs) published between January 1996 to November 2025 by using predefined search terms: (vitamin B OR pyridoxine OR pyridoxal OR pyridoxamine OR folate OR folic acid OR cobalamin OR B vitamins) AND (homocysteine OR homocysteinemia OR hyperhomocysteinemia) AND (thrombosis OR thrombotic OR cardiovascular event OR stroke OR unstable angina OR myocardial infarction OR cardiovascular accident OR thromboembolism). See Figure 1.

### 2.2. Eligibility Criteria

A total of thirteen clinical trials were included in this meta-analysis. Randomized controlled trials were eligible if they met the following criteria:

Population: adult patients (greater than or equal to 18 years of age).

Duration: minimal follow-up duration greater than 24 months.

Intervention: oral, enteral, or parenteral folic acid (Vitamin B9) and/or cobalamin (or vitamin B12) and/or pyridoxine (vitamin B6).

Control group: the control group received either a placebo or doses of B vitamins lower than the recommended daily doses.

Outcomes: Incidence of any arterial thrombotic events, including (but not limited to) unstable angina, myocardial infarction (MI), stroke or transient ischemic attack (TIA), cardiovascular accident (CVA), all-cause mortality, and cardiovascular disease mortality.

### 2.3. Study Selection and Quality Assessment

Two authors independently reviewed the study design and methodological quality of all included trials. Discrepancies were resolved by discussion or consultation with a third author.

### 2.4. Definitions

Due to variability in reporting across the included studies, standardized definitions were applied for this meta-analysis.

Prevention type was defined based on the study population: the trials enrolling only participants without pre-existing cardiovascular disease were categorized as primary prevention; those enrolling only patients with established cardiovascular disease were classified as secondary prevention; and those including both primary and secondary prevention participants were classified as mixed.

The estimated risk of coronary heart disease (CHD) was defined based on the characteristics of each study population, based on the 2018 AHA/ACC guidelines [9]. Participants were classified as high risk if the majority of enrolled patients had a documented history of coronary artery disease (CAD), other vascular disease (including kidney transplant recipients), diabetes mellitus, prior stroke or transient ischemic attack (TIA), or an estimated 10-year ASCVD risk > 20%. Trials enrolling predominantly primary prevention populations or participants with an estimated 10-year ASCVD risk < 7.5% were classified as low risk.

The composite outcome of 3-point major adverse cardiovascular events (MACE) was regarded as the combined occurrence of cardiovascular death, nonfatal myocardial infarction, or nonfatal stroke.

### 2.5. Data Extraction

Data extraction was initially performed by one of the authors and independently verified by two other authors. Discrepancies were resolved through group discussion and consensus. Attempts were made to contact the original study authors to obtain the raw and supplemental data; however, due to the lack of responses, only data available from published reports were included in this analysis.

The following study characteristics were extracted: patient population and baseline characteristics, atherosclerotic cardiovascular disease (ASCVD) risk, baseline comorbidities, duration of follow-up, home medication use, antiplatelet use, intervention, outcomes, and subgroup analyses.

### 2.6. Statistical Analysis

Statistical analysis was performed using the meta package version 8.1-0 and R version 4.5.0 [10]. Risk ratios using a Mantel–Haenszel (MH) random-effects meta-analysis were calculated to combine the studies. A MH random-effects model was chosen because RCTs varied in baseline vitamin exposure, patient populations, and dosing regimens. Statistical heterogeneity was assessed using the I^2^ statistic, Cochran’s Q statistic (and respective *p*-value), and τ^2^. A forest plot was used to display the individual and pooled effect estimates, including the 95% confidence intervals [11]. The risk of bias for the enrolled randomized controlled trials was assessed using the Cochrane Risk of Bias 2 (RoB 2) tool [12], applied separately for the all-cause mortality, stroke and MI outcomes.

## 3. Results

A total of 13 studies involving 68,363 participants were included in this meta-analysis. The baseline characteristics of these studies, including sample sizes, participant description, primary versus secondary prevention, estimated ASCVD scores of participants, median follow-up durations, intervention and control group, doses, and outcomes, are summarized in Table 1.

**Figure 2:**
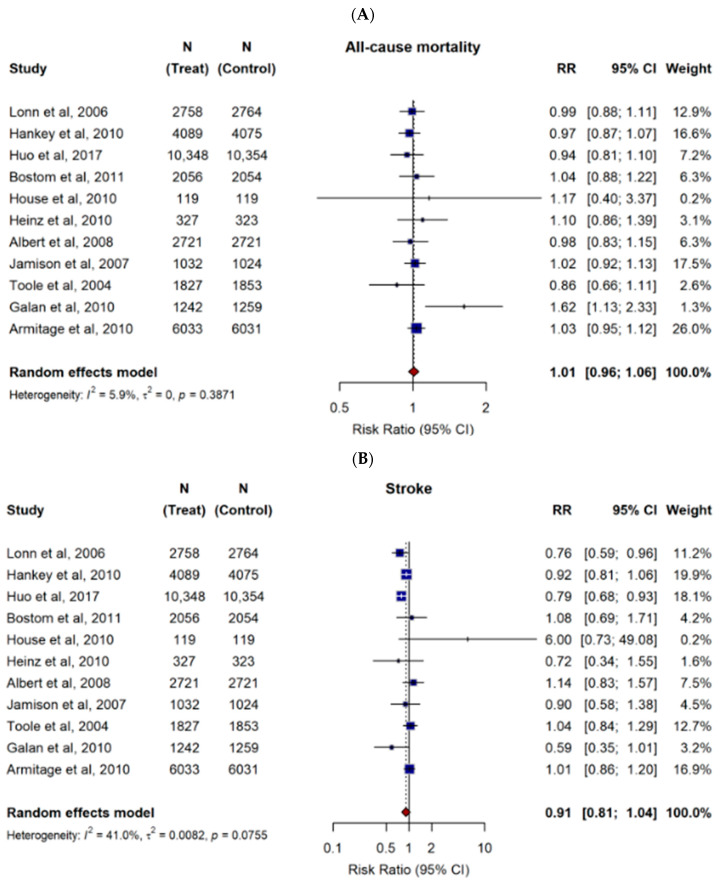

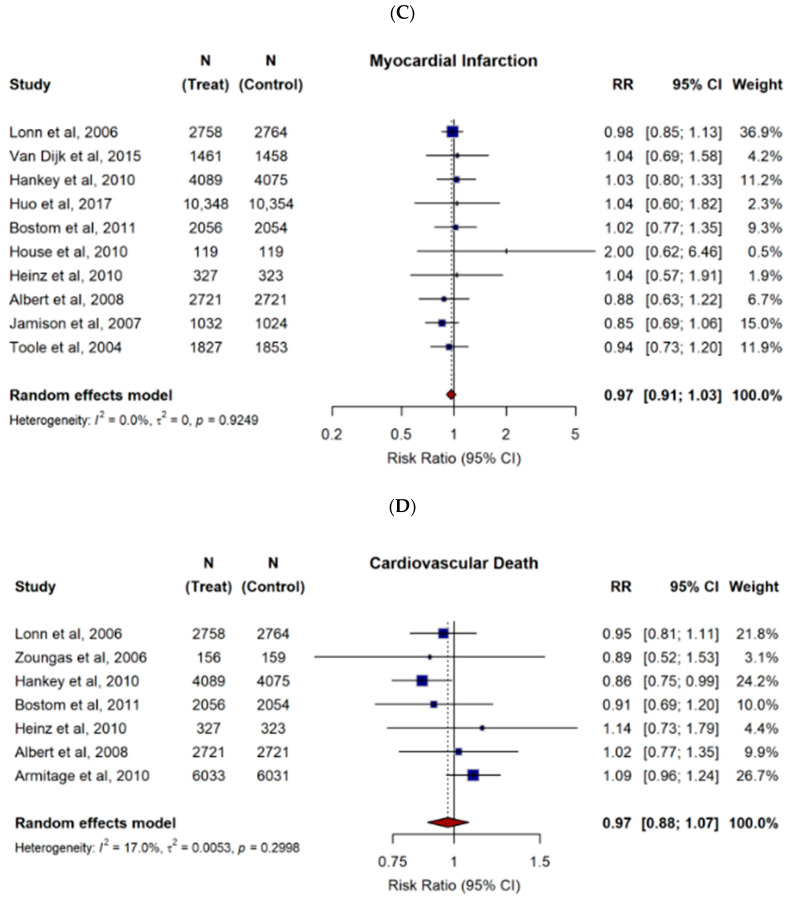

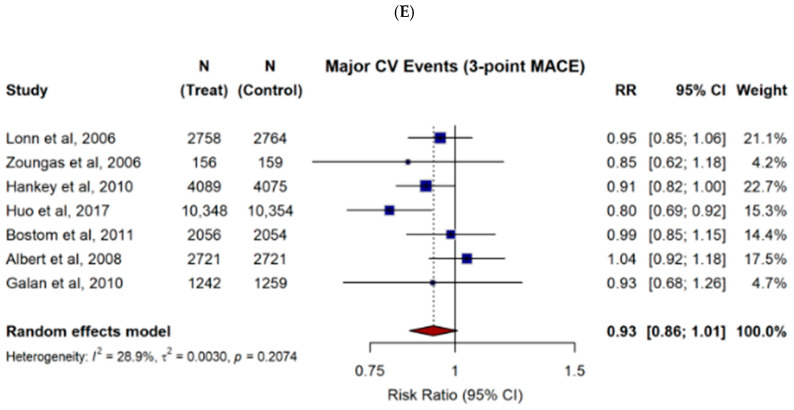
Pooled estimates of risk ratios (RRs) for the effect of B-vitamin combinations versus placebo on major clinical outcomes. Forest plots display pooled and individual study estimates for (**A**) all-cause mortality, (**B**) stroke, (**C**) myocardial infarction (MI), (**D**) cardiovascular death, and (**E**) major adverse cardiovascular events (3-point MACE). Effect sizes were calculated using a random-effects model. Horizontal lines indicate 95% confidence intervals. The size of each square represents the weight of the corresponding study in the meta-analysis. The diamond represents the pooled estimate of the overall results. The solid vertical line represents the line of no effect (RR = 1). The dotted vertical line represents the pooled overall effect estimate of all included studies.

The results from random-effects models pooling the risk ratios (RR) for stroke, myocardial infarction (MI), cardiovascular death, all-cause mortality, and 3-point MACE (major adverse cardiovascular events) are demonstrated in Figure 2. Our meta-analysis showed that B-vitamin combinations were associated with a downward trend in the risk of stroke and 3-point MACE, though not significant (RR = 0.91, 95% CI: 0.81–1.04, *p* = 0.14; RR = 0.93, 95% CI: 0.86–1.01, *p* = 0.073, respectively). As for all-cause mortality, cardiovascular death, and MI, B-vitamin combinations did not result in significant changes (RR = 1.01, 95% CI: 0.96–1.07, *p* = 0.61; RR = 0.97, 95% CI: 0.88–1.07, *p* = 0.50; RR = 0.97, 95% CI: 0.91–1.03, *p* = 0.24, respectively). To evaluate data quality and confidence in the conclusions, the summary of each clinical outcome by the number of trials, number of participants, event counts, and pooled relative risks (RRs) with 95% confidence intervals (CIs) is outlined in Appendix A Table A1.

### 3.1. Risk of Bias

**Figure 3:**
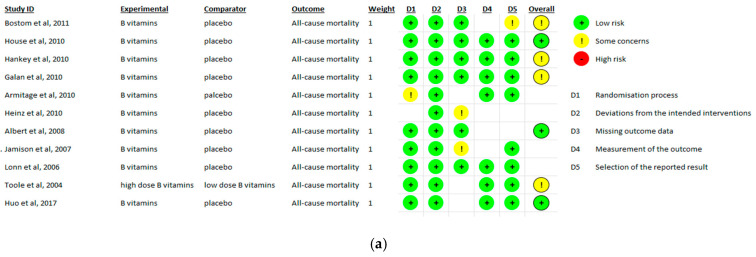

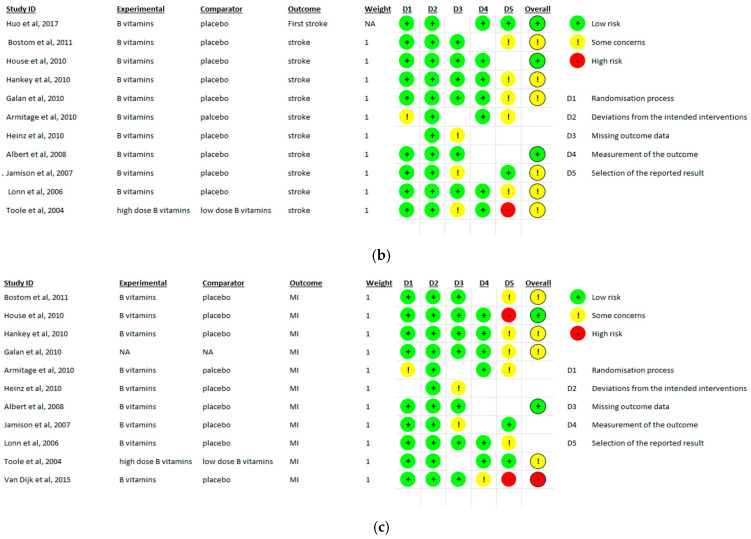
(**a**) Risk of bias assessment for all-cause mortality outcomes using the Cochrane Risk of Bias 2 (RoB 2) tool. (**b**) Risk of bias assessment for stroke outcomes using the Cochrane Risk of Bias 2 (RoB 2) tool. (**c**) Risk of bias assessment for myocardial infarction (MI) outcomes using the Cochrane Risk of Bias 2 (RoB 2) tool.

**Figure 4:**
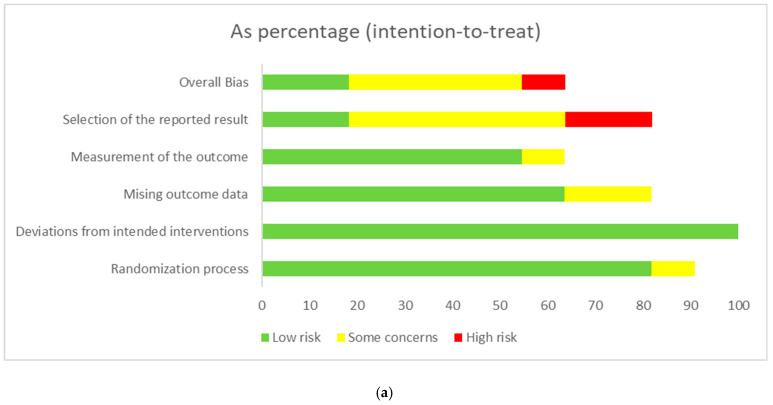

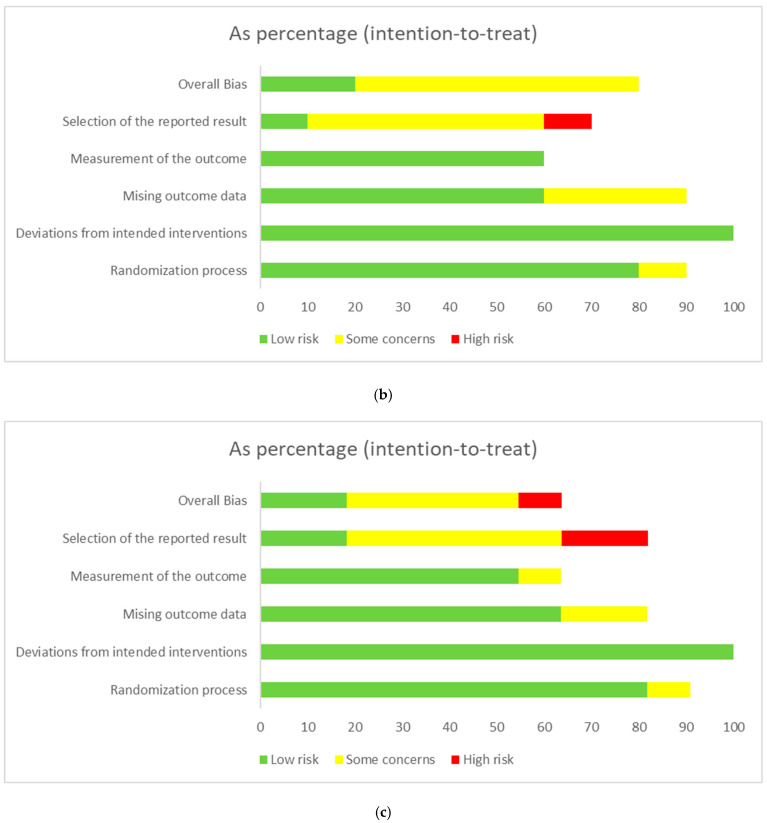
(**a**) Pooled quantitative risk of bias assessment for all-cause mortality outcomes. (**b**) Pooled quantitative risk of bias assessment for stroke outcomes. (**c**) Pooled quantitative risk of bias assessment for myocardial infarction outcomes.

Eleven studies were included for bias risk analysis for the all-cause mortality, stroke and MI outcomes, respectively. Most trials were judged to be at low risk of bias in the area of randomization process, minimal deviation from the intended intervention, minimal missing data, and appropriate measurement of the outcomes. Several studies showed some concerns of high risk of bias, largely attributable to heterogeneous outcome definitions, indirectness, and selective reporting of outcome measurement. The evidence of risks of bias for the included studies is summarized in Figure 3a–c, with the corresponding graphical representations shown in Figure 4a–c.

### 3.2. Subgroup Analysis

#### 3.2.1. Primary Versus Secondary Prevention of Cardiovascular Disease

**Figure 5:**
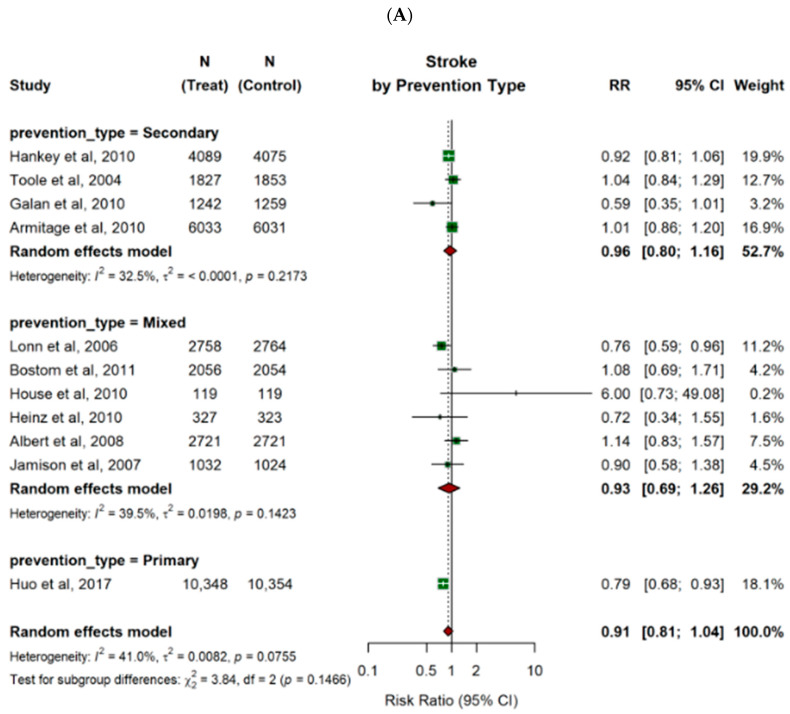

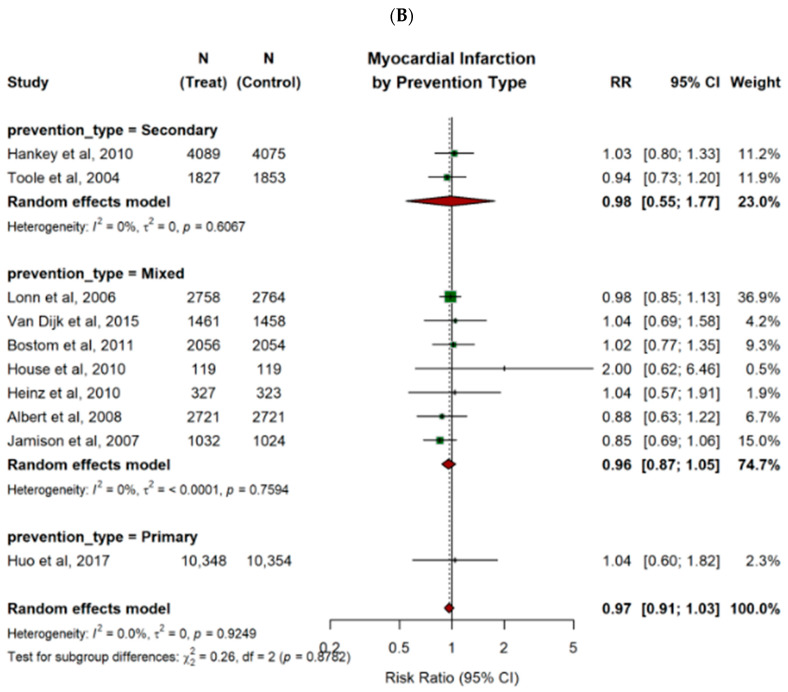

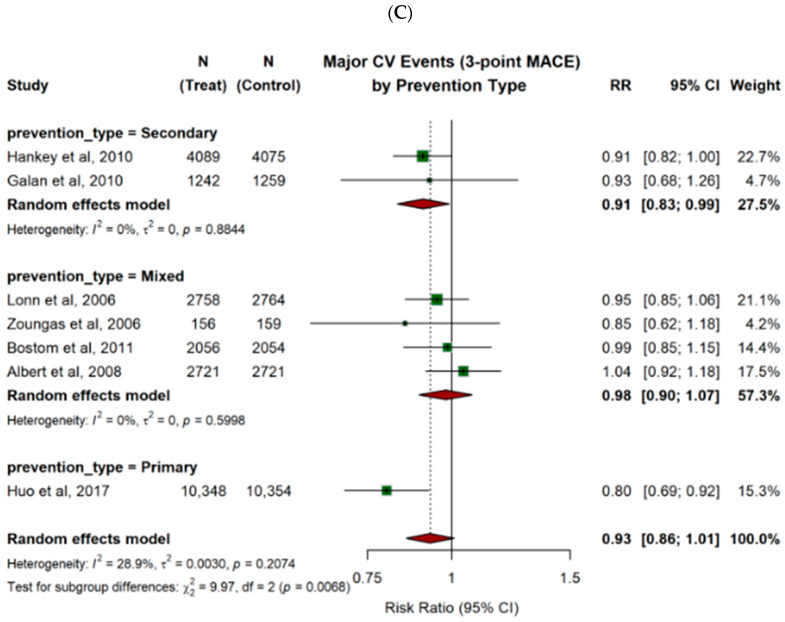
Pooled estimates of risk ratios (RRs) for the effect of B-vitamin combinations versus control group on major clinical outcomes, stratified by the prevention type of the study populations (secondary prevention, mixed primary and secondary prevention, and primary prevention alone). Forest plots display pooled and individual study estimates for (**A**) stroke and (**B**) MI, and (**C**) 3-point MACE. Effect sizes were calculated using a random-effects model. Horizontal lines indicate 95% confidence intervals. The size of each square represents the weight of the corresponding study in the meta-analysis. The diamond represents the pooled estimate of the overall results. The solid vertical line represents the line of no effect (RR = 1). The dotted vertical line represents the pooled overall effect estimate of all included studies.

B-vitamin combinations were associated with a significant reduction in the risk of stroke (RR 0.79; 95% CI 0.68–0.93) and MACE (RR 0.80; 95% CI 0.69–0.92) for primary prevention of cardiovascular disease. It also showed a small, but significant, decrease in risk of MACE for secondary prevention of cardiovascular disease (RR 0.91; 95% CI 0.83–0.99). Other pooled estimates did not demonstrate significant effects (as shown in Table 2 and Figure 5).

Heterogeneity was minimal to low for the majority of outcomes. However, the all-cause mortality and cardiovascular mortality in the secondary prevention subgroup demonstrated significant heterogeneity (I^2^ = 66.7%, *p* = 0.029 and I^2^ = 83.8%, *p* = 0.013, respectively).

#### 3.2.2. Patients with High Risk Versus Low Risk of ASCVD

**Figure 6:**
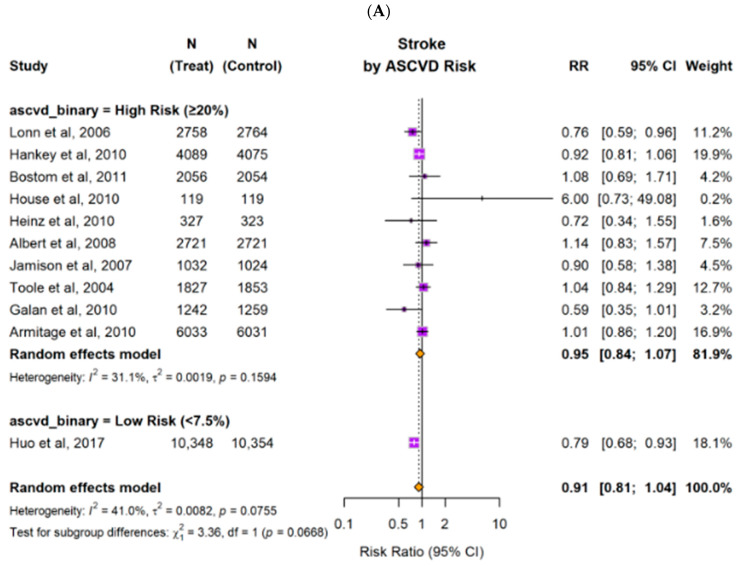

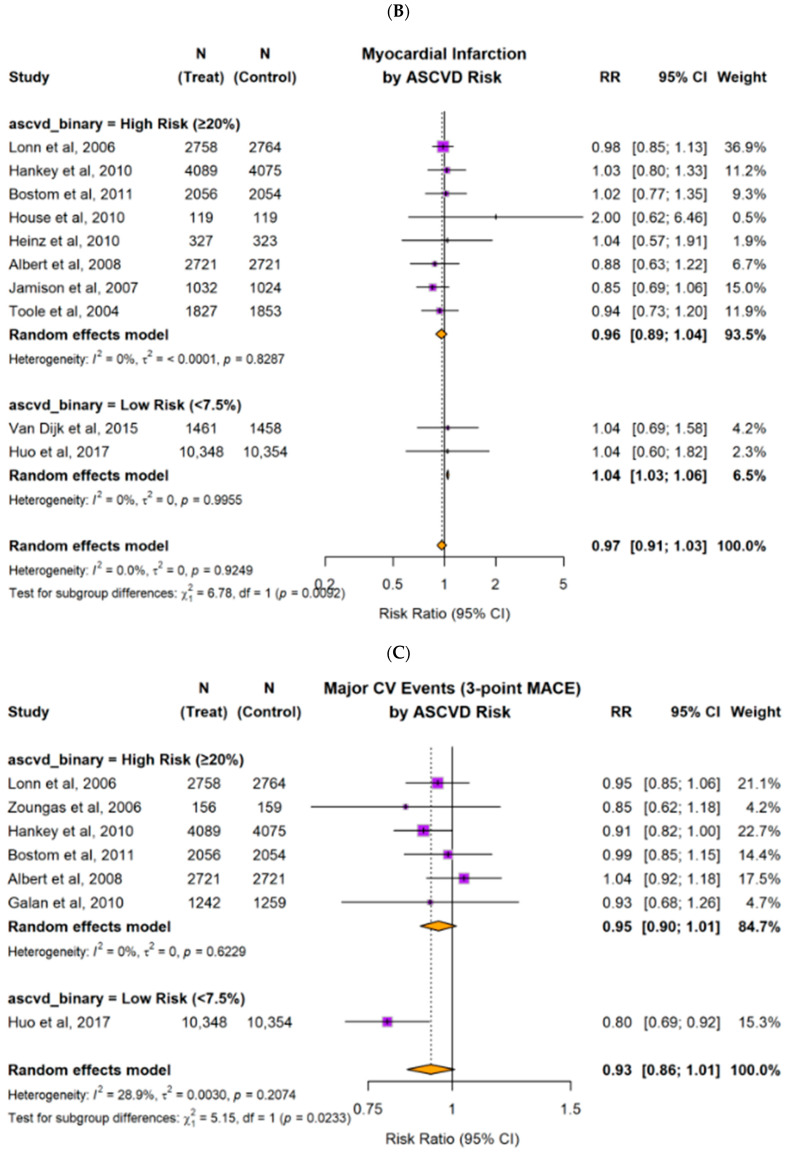
Pooled estimates of risk ratios (RRs) for the effect of B-vitamin combinations versus control group on major clinical outcomes, stratified by the ASCVD risk (high- versus low-risk). Forest plots display pooled and individual study estimates for (**A**) stroke, (**B**) myocardial infarction (MI), and (**C**) major adverse cardiovascular events (3-point MACE). Effect sizes were calculated using a random-effects model. Horizontal lines indicate 95% confidence intervals. The size of each square represents the weight of the corresponding study in the meta-analysis. The diamond represents the pooled estimate of the overall results. The solid vertical line represents the line of no effect (RR = 1). The dotted vertical line represents the pooled overall effect estimate of all included studies.

Subgroup analyses were performed for patients with high and low ASCVD risk (Figure 6). For patients with high ASCVD risk, B-vitamin combinations resulted in no significant effect across cardiovascular outcomes. The relative risk was 0.95 (95% CI: 0.84–1.07) for stroke, 0.96 (95% CI: 0.89–1.04) for MI, and 0.95 (95% CI: 0.90–1.01) for 3-point MACE. All confidence intervals crossed 1.0, indicating no statistically significant benefit in this subgroup. For patients with low ASCVD risk, B-vitamin combinations were associated with lower risk of 3-point MACE as well as stroke (RR 0.8 (0.69–0.92), *p* = 0.0068 and RR 0.79 (0.68–0.93), respectively, but higher MI risk (RR 1.04 (1.03–1.06), *p* < 0.01).

## 4. Discussion

The role of B-vitamin combinations in reducing stroke and cardiovascular events through homocysteine lowering has been debated for decades. Previous studies have yielded inconsistent results. It was suggested that folic acid alone may reduce stroke risk, particularly in countries without mandatory folic acid fortification, whereas combining folic acid with vitamins B6 and B12 does not consistently improve outcomes [25,26,27]. Differences in study populations, folic acid fortification policies, and trial designs likely contribute to the variability in reported effects. Moreover, optimal dosing remains uncertain: folic acid has been studied at 0.8–2.5 mg/day, and vitamin B12 at 0.05–0.4 mg/day [28,29].

Recent meta-analyses have provided further insights. Miao et al. (2024) concluded that folic acid can reduce carotid intima-media thickness and that B-vitamin combinations may lower cardiovascular disease risk in patients with normal renal function and without recent unstable angina or non-ST elevation myocardial infarction [30], indicating B-vitamin combinations with possible primary prevention benefit. Conversely, the study by Bønaa et al. (2006) in 3749 post-MI patients found no benefit of B-vitamin combination supplementation and suggested potential harm from the combination therapy [31] in secondary prevention populations. Wang et al. in 2015 reported reductions in serum homocysteine, stroke recurrence, MI, and vascular death in a meta-analysis of 7474 patients [32], while Zhang et al. in 2024 demonstrated that in regions without or with partial folic acid fortification, B-vitamin combinations reduced stroke risk by 34%, with optimal doses of folic acid < 0.8 mg/day and B12 < 0.4 mg/day [33]. No benefit was observed in fortified regions [32,33]. Inconsistent study design of the trials, poorly defined patient populations and non-standard clinical outcomes made it difficult for a meaningful clinical conclusion.

Our meta-analysis expands on previous studies by evaluating not only stroke but also MI, cardiovascular death, 3-point MACE, and all-cause mortality. Additionally, we performed subgroup analyses stratified by primary versus secondary prevention and baseline ASCVD risk. To our knowledge, this is the first comprehensive meta-analysis encompassing all these outcomes. Our findings indicate that B-vitamin combinations had no significant effect on all-cause mortality, stroke, MI, cardiovascular death, or major cardiovascular events. The effects were similar in patients with or without end-stage renal disease. Interestingly, in regions with voluntary folic acid fortification, B-vitamin combinations supplementation appeared harmful overall, although it showed potential benefits for cardiovascular death. Subgroup analyses suggested a reduction in 3-point MACE in primary prevention populations and in patients with low ASCVD risk, but paradoxically, MI incidence was higher in the B-vitamin combination group. These subgroup findings, however, should be considered as hypothesis-generating and interpreted with caution given the relatively small sample size and strong influence from single large studies.

These findings highlighted the inconsistencies in the literature regarding B-vitamin combination supplementation and cardiovascular outcomes. Large trials such as NORVIT, HOPE-2, and VISP have shown that while B vitamins effectively reduce serum homocysteine, they do not reduce rates of MI, stroke, or cardiovascular death. Supplementation appears most beneficial in cases of true folate or B12 deficiency or genetic homocystinuria (cystathionine beta-synthase deficiency). Overdose is not recommended; high-dose B6 (>200 mg/day) may cause neuropathy, and high-dose folate can mask B12 deficiency [34].

Our results suggest that baseline cardiovascular risk may influence the benefit of B- vitamin combinations. Patients with low baseline CVD risk appeared to derive greater benefit in reducing stroke and major cardiovascular events, whereas those with established CVD, high ASCVD risk (>20%), or advanced renal disease showed minimal benefit. This aligns with previous findings indicating that in patients with advanced atherosclerosis, the vascular and oxidative stress damage associated with prolonged hyperhomocysteinemia, may be unresponsive to B-vitamin therapy [35,36,37].

B-vitamin combinations might have a role in primary prevention of arterial thrombotic events, particularly in low-risk populations, while their utility in secondary prevention or high-risk patients is limited. Future research should focus on identifying patient populations most likely to benefit, considering baseline cardiovascular risk, homocysteine levels, folate/B12 status, and regional fortification practices.

### 4.1. Patients with End-Stage Renal Disease

Patients with advanced kidney disease exhibit higher plasma homocysteine levels and an increased risk of cardiovascular diseases than patients without advanced kidney disease [38]. Combined with the findings that B-vitamin combinations reduce serum homocysteine level [39], this has led to the assumption that the addition of B vitamins may produce a protective role in cardiovascular disease in this population. Our exploratory analyses in patients with end-stage renal disease similarly did not provide any significant benefit in terms of stroke, myocardial infarction, or improving survival with the supplementation of B-vitamin combinations. These findings indicate that, although B-vitamin therapy effectively lowers plasma homocysteine in patients with renal disease, it has not been shown to reduce the risk of major cardiovascular events [40,41]. The lack of a significant clinical benefit with B vitamins may reflect the advanced vascular lesions in patients with end-stage renal disease, which may limit the beneficial effects of lowering homocysteine. Variations in baseline homocysteine levels, differences in dialysis regimens, and the relatively small sample sizes may also help explain these findings.

Furthermore, the inability to demonstrate a mortality benefit with B-vitamin combinations in ESRD may parallel findings from the use of statins in ESRD trials, where initiating statins in dialysis patients improved LDL but failed to improve survival [42,43]. In both cases, the high baseline mortality and extensive comorbid burden in patients with end-stage renal disease may overshadow the potential benefits of these interventions.

### 4.2. Fortification of Folic Acid

In this meta-analysis, B-vitamin combinations are associated with increased harm in voluntary folic acid fortification areas for MI prevention. On the contrary, vitamin B supplement is also demonstrated to be effective in CV death rate in areas with voluntary folic acid fortification. Zhang et al. in 2024 performed a meta-analysis, which found that vitamin B supplementation significantly reduced the risk of stroke in areas without and with partial folic acid fortification [33]. Unfortunately, this finding is not seen in our exploratory analyses and needs further confirmation.

### 4.3. ASCVD Risk

For patients with low ASCVD risk, the effects of B-vitamin combinations are limited and largely based on a small number of contributing studies. The observed reduction in stroke risk (RR, 0.78; 95% CI, 0.68–0.93) and in 3-point major adverse cardiovascular events (RR, 0.80; 95% CI, 0.69–0.92) were driven entirely by the CSPPT trial, conducted in a setting without mandatory folic acid fortification [13]. Because of the very low incidence of stroke and 3-point MACE in the primary prevention low-risk group, large sample sizes are required to achieve adequate statistical power; approximately 15,000 participants for stroke outcomes and more than 12,000 participants for 3-point MACE are typically needed to reach 80–90% power. The CSPPT trial alone did not meet these thresholds for detecting modest effect sizes. In contrast, the myocardial infarction estimate was derived from two trials and demonstrated a statistically significant increase (RR, 1.04; 95% CI, 1.03–1.06). The estimate for MI risk came from two trials [13,14] and showed a statistically significant increase, indicating a potential signal of harm in this subgroup. Even though statistically significant, an observed risk ratio of 1.04 reflects a very small effect and may lack clinical relevance.

### 4.4. Strengths and Limitations

Our meta-analysis has several key strengths, including restriction to randomized controlled trials with at least 24 months of follow-up, exclusion of multivitamin formulations to minimize confounding, and use of rigorous methodology with independent data extraction, prespecified subgroup analyses, and random-effects modeling. The evaluation of standardized cardiovascular outcomes—including all-cause mortality, stroke, myocardial infarction, cardiovascular death, and 3-point major adverse cardiovascular events—together with stratification by prevention setting, baseline ASCVD risk, renal function, and folic acid fortification practices, enabled assessment of potential context-dependent effects. Notably, this study is among the first to examine differential associations of B-vitamin combinations with 3-point MACE across cardiovascular risk strata while confirming neutral effects in higher-risk populations.

These strengths should be interpreted in light of important limitations. Included trials varied in participant characteristics, baseline cardiovascular risk, B-vitamin dosing and combinations (e.g., folic acid alone vs. combined B-vitamin regimens), outcome definitions, and follow-up duration, which may have contributed to heterogeneity and limited comparability. Subgroup analyses were based on trial-level classifications and, in some cases, a limited number of contributing studies; therefore, these findings should be considered exploratory and hypothesis-generating and may be subject to ecological bias. In addition, reliance on aggregate data precluded adjustment for individual-level factors such as baseline homocysteine concentration, vitamin B12 status, genetic variation, dietary intake, and concomitant therapies, and adverse events were not consistently reported across trials. Overall certainty was highest for ischemic outcomes in primary prevention, supported by low heterogeneity and consistent direction of effect, but remained low for mortality outcomes owing to imprecision and substantial between-study variability. Finally, publication bias and small-study effects cannot be excluded, and strict eligibility criteria may limit generalizability to broader real-world populations.

## 5. Conclusions

Overall, our comprehensive meta-analysis demonstrated that B-vitamin combinations do not offer benefit for major cardiovascular outcomes. The effect on the reduction of stroke and MACE in selected primary prevention populations needs to be confirmed in future studies.

## Figures and Tables

**Table 1:** Baseline characteristics of the studies included in the meta-analysis.

Author/Year	Participants	Other Baseline Comorbidities	Primary vs. Secondary Prevention	Estimated ASCVD Score	Follow-Up Duration	Intervention and Dose
1. Huo et al., 2015 [13]	20,702 adults with hypertension without a history of stroke or MI	Hyperlipidemia (2.7%), diabetes (3.1%)	Primary	Low ASCVD risk < 7.5% risk	4.5 years	Folic acid 0.8 mg vs. placebo
2. van Dijk et al., 2015 [14]	2919 elderly aged at least 65 years with hyperhomocysteinemia (12–50 μmol/L)	Any type of CVD history (19%), MI (7%), hypercholesterolemia (19%), cerebrovascular event (7%), diabetes (8%)	Mixed	Low ASCVD risk, <7.5% risk	3.4 years	Folic acid 0.4 mg, vitamin B12 0.5 mg vs. placebo
3. Bostom et al., 2006 [15]	4110 stable kidney transplant recipients	History of CVD (20%), diabetes (40%), hypertension (92%)	High risk/Mixed	High-risk, ASCVD 20% or higher	4 years	Folic acid 5.0 mg, vitamin B6 50 mg, and vitamin B12 1.0 mg vs. MVI with B6 1.4 mg, B12 2.0 mcg, and no folic acid
4. House et al., 2010 [16]	238 participants who had type 1 or 2 diabetes and a clinical diagnosis of diabetic nephropathy	Hypertension (94%), hyperlipidemia (85%), MI or angina (31%), stroke or TIA (14.3%), peripheral vascular disease (17%)	High risk/Mixed	High-risk, ASCVD 20% or higher	3 years	Folic acid (2.5 mg/d), vitamin B6 (25 mg/d), and vitamin B12 (1 mg/d) vs. placebo
5. Hankey et al., 2013 [17]	8164 patients with recent stroke or TIA (within the past 7 months)	History of stroke (16%), MI (7%), ischemic heart disease (17%), history of hypertension (71%), ischemic heart disease (17%)	Secondary	High-risk, ASCVD 20% or higher	2 years	Folic acid 2 mg, vitamin B6 25 mg, and vitamin B12 0.5 mg vs. placebo
6. Galan et al., 2010 [18]	2501 patients with a history of MI, UA, or ischemic stroke	History of MI (46%), unstable angina (28.5%), stroke (25.5%)	Secondary	High-risk, ASCVD 20% or higher	4.7 years	5-methyl-THF 0.56 mg, vitamin B6 3 mg, vitamin B12 0.02 mg vs. placebo
7. Armitage et al., 2010 [19]	12,064 survivors of MI	History of MI (48%), MI + other CHD (42%), DM (11%), other vascular disease (9%)	Secondary	High-risk, ASCVD 20% or higher	6.7 years	Folic acid 2 mg, vitamin B12 1 mg vs. placebo
8. Heinz et al., 2010 [20]	650 patients with end-stage renal disease who were undergoing hemodialysis	Hypertension (89%), DM (40%)	High risk/Mixed	High-risk, ASCVD 20% or higher	6 years	Active treatment: folic acid 5 mg, vitamin B12 50 μg, and vitamin B6 20 mg given 3 times a week; Placebo: folic acid 0.2 mg, vitamin B12 4 μg, and vitamin B6 1.0 mg, given 3 times a week
9. Albert et al., 2008 [21]	5442 female US health professionals aged 40 years or older with either a history of CVD or ≥three coronary risk factors	Prior CVD (64.1%), risk factors (86.2%)-hypertension, elevated cholesterol, DM, current smoking, parental history of MI, BMI ≥ 30 kg/m^2^, (at least weekly) alcohol intake	High risk/Mixed	High-risk, ASCVD 20% or higher	7.3 years	Folic acid 2.5 mg, vitamin B6 50 mg, and vitamin B12 1 mg daily vs. placebo
10. Jamison et al., 2007 [22]	2056 patients with advanced CKD (eCrCl 30 mL/min) or ESRD, and high Hcy levels (≥15 µmol/L).	MI (25%), CHF (23.5%), hypertension (96%), angina (25%), PCI or stenting (14%), CABG (18%), stroke (15%), DM (55%)	High risk/Mixed	High-risk, ASCVD 20% or higher	3.2 years	Folic acid 40 mg, vitamin B6 100 mg, and vitamin B12 2 mg daily vs. placebo
11. Lonn et al., 2006 [6]	5522 patients with vascular disease or diabetes	CAD (83.3%), MI (54.3%), unstable angina (26.1%), CABG (27.2%), PCI (20.2%), stroke or TIA (12.4%)	High risk/Mixed	High-risk, ASCVD 20% or higher	5 years	Folic acid 2.5 mg, vitamin B6 50 mg, and vitamin B12 1.0 mg vs. placebo
12. Zoungas et al., 2006 [23]	315 patients, aged 24–79 years, chronic renal failure	Hypertension (90%), diabetes (23%), hypercholesterolemia (44%), pre-existing cardiovascular disease (35%)	High risk/Mixed	High-risk, ASCVD 20% or higher	3.6 years	Folic acid 15 mg vs. placebo
13. Toole et al., 2004 [24]	3680 adults with nondisabling cerebral infarction	Hypertension (73.7%), diabetes (29.1%), any cardiac disease (24.6%), stroke history (23.3%), angina (7.4%)	Secondary	High-risk, ASCVD 20% or higher	2 years	High-dose group (vitamin B6 25 mg, vitamin B12 0.4 mg, and folic acid 2.5 mg daily); low-dose group (vitamin B6 200 μg, vitamin B12 6 μg, and folic acid 20 μg daily)

**Table 2:** Pooled risk ratios (RR) for all outcomes were estimated using a random-effects model, stratified by type of prevention (secondary vs. mixed vs. primary). MI: myocardial infarction; MACE: major adverse cardiovascular events, including all-cause mortality, stroke, and MI; 95% CI: 95% confidence interval; NR: heterogeneity (I^2^) not reported, only one study included.

Outcomes	Stratified by Prevention Type	No. of Patients	RR	95% CI	Heterogeneity, *p*-Value
All-cause mortality	Secondary prevention	26,409	1.04	0.74; 1.50	I^2^ = 66.7%, *p* = 0.0291
	Mixed	18,018	1.01	0.98; 1.05	I^2^ = 0%, *p* = 0.9709
	Primary prevention	20,702	0.94	0.81; 1.10	NR
Stroke	Secondary prevention	26,409	0.96	0.80; 1.16	I^2^ = 32.5%, *p* = 0.2173
	Mixed	18,018	0.93	0.69; 1.26	I^2^ = 39.5%, *p* = 0.1423
	Primary prevention	20,702	0.79	0.68; 0.93	NR
MI	Secondary prevention	11,844	0.98	0.55; 1.77	I^2^ = 0%, *p* = 0.6067
	Mixed	20,937	0.96	0.87; 1.05	I^2^ = 0%, *p* = 0.7594
	Primary prevention	20,702	1.04	0.60; 1.82	NR
Cardiovascular death	Secondary prevention	20,228	0.97	0.21; 4.48	I^2^ = 83.8%, *p* = 0.0013
	Mixed	16,039	0.96	0.89; 1.05	I^2^ = 0%, *p* = 0.9102
MACE	Secondary prevention	10,665	0.91	0.83; 0.99	I^2^ = 0%, *p* = 0.8844
	Mixed	15,389	0.98	0.90; 1.07	I^2^ = 0%, *p* = 0.5998
	Primary prevention	20,702	0.80	0.69; 0.92	NR

## Data Availability

No new data were generated in this study.

## Appendix A

**Table A1:** Association of B-vitamin combinations with major cardiovascular outcomes. Relative risks (RRs) and 95% confidence intervals (CIs) were pooled using a random-effects model. Statistical heterogeneity was assessed using the I^2^ statistic and the Cochrane Q test. Het P represents the *p*-value of heterogeneity.

Outcome	No. of Trials	Participants	Events (Treatment)	Events (Control)	RR (95% CI)	*p* Value	I^2^ (%)	Het *P*
All-cause mortality	11	65,129	3598/32,552 (11.1%)	3573/32,577 (11.0%)	1.01 (0.96–1.06)	0.747	6	0.387
Stroke	11	65,129	1366/32,552 (4.2%)	1500/32,577 (4.6%)	0.91 (0.81–1.04)	0.14	41	0.08
Myocardial infarction	10	53,483	961/26,738 (3.6%)	994/26,745 (3.7%)	0.97 (0.91–1.03)	0.24	0	0.93
Cardiovascular death	7	36,267	1312/18,140 (7.2%)	1344/18,127 (7.4%)	0.97 (0.88–1.07)	0.50	17	0.30
Major CV events (3-point MACE)	7	46,756	2276/23,370 (9.7%)	2451/23,386 (10.5%)	0.93 (0.86–1.01)	0.07	29	0.21
